# Supplementary material for: Dog Walking before and during the COVID-19 Pandemic Lockdown: Experiences of UK Dog Owners
Source: Int J Environ Res Public Health. 2021 Jun 10;18(12):6315. doi: 10.3390/ijerph18126315 (PMC8296116; doi:10.3390/ijerph18126315)
Supplement: Supplementary file 1 [file ijerph-18-06315-s001.zip › ijerph-1203471-supplementary.pdf]

Supplementary material file

Table S1: Multivariable regression analysis of variables associated with duration of dog's walking before the first UK COVID-19 lockdown (March / April 2020). Model based on 510 observations. F(22) = 5.38, *P* = 0.00, Cragg-Uhler Pseudo-R2 =0.28, AIC=243.18**.**

Variable	Regression coefficient	Standard Error	t value	2.5% CI	97.5% CI	*P*
**Intercept**	2.31	0.13	18.12	2.04	2.75	< 2e-16
Number of dogs: 1	0					
Number of dogs: 2	-0.03	0.03	-0.83	-0.16	0.02	0.41
Number of dogs: 3	-0.05	0.04	-1.10	-0.15	0.09	0.27
Dog size: small/ toy	0					
Dog size: medium	0.04	0.04	1.08	-0.05	0.14	0.28
Dog size: large/giant	0.09	0.04	2.45	-0.05	0.16	0.01
Age: Under 1	0					
Age: 1-5	0.00	0.06	-0.02	-0.20	0.14	0.99
Age: 6-10	0.03	0.06	0.51	-0.15	0.21	0.61
Age: 10+	0.01	0.08	0.07	-0.23	0.21	0.94
Dog's sex: female	0					
Dog's sex: male	-0.02	0.03	-0.78	-0.07	0.08	0.43
Dog's energy: low	0					
Dog's energy: medium	0.09	0.06	1.70	-0.07	0.24	0.09
Dog's energy: high	0.24	0.06	4.02	0.03	0.35	<0.001
Relationship: weak	0					
Relationship: medium	0.02	0.09	0.17	-0.24	0.25	0.86
Relationship: strong	0.01	0.09	0.14	-0.27	0.22	0.89
Owner does not live alone	0					
Owner lives alone	0.04	0.04	1.11	-0.05	0.16	0.27
Owner's age: 18-30	0					
Owner's age: 30-50	0.09	0.04	2.47	-0.04	0.17	0.01
Owner's age: 50+	0.10	0.04	2.42	0.01	0.23	0.02
Owner's gender: male	0					
Owner's gender: female	0.07	0.04	1.51	-0.08	0.17	0.13
Owner's education: university Degree	0					
Owner's education: professional	0.00	0.04	0.07	-0.10	0.11	0.94
Owner's education: A-level or equivalent	-0.11	0.04	-2.52	-0.23	0.01	0.01
Owner's education: below A-level or equivalent	-0.01	0.04	-0.14	-0.26	-0.01	0.89

Table S2**:** Weekly duration of dog's and person's walking before and during the COVID-19 first national lockdown. Within group comparison (change in walking duration between before and during the lockdown) is summarised with sample size (n), Mann-Whitney paired test-statistic (V) and significance levels (*P*).

**Variable**	**Dog's walking**		**Within-group change-dogs**	**Person's walking**		**Within-group change-people**
	**Before the lockdown,**	**During the lockdown,**		**Before the lockdown,**	**During the lockdown,**	

	median (IQR)	median (IQR)		median (IQR)	median (IQR)	
Weekly duration	420 (350)	420 (330)	539, V= 136373, P=0.41	350 (360)	360 (270)	526, V=135915, P=0.62
Living arrangements: Living alone	500 (500)	420 (330)	82, V=2849, P= 0.09	440 (420)	420 (335)	82, V=3093, P=0.38
Living with others	420 (350)	420 (300)	443, V=97306, P=0.87	300 (300)	315 (210)	435, V=9426, P=0.93
Household composition: Single-dog household	420 (397.5)	420 (330)	300, V=44446, P=0.79	300 (300)	325 (214.25)	295, V=44278, P=0.71
2 dogs	420 (320)	420 (310)	157, V=11853, P=0.62	400 (360)	365 (285)	159, V=1227, P=0.66
3+ dogs	420 (387.5)	420 (335)	73, V=2445, P=0.39	420 (405)	420 (350)	72, V=2207, P=0.12
Dog size: Toy/ Small	420 (290)	420 (285)	118, V=7320, P=0.49	300 (315)	345 (220)	121, V=7545, P=0.68
Medium	420 (350)	420 (350)	220, V=25335, P=0.35	360 (367)	400 (210)	219, V=23066, P=0.49
Large or Giant	467.5 (400)	420 (330)	188, V=16937, P= 0.49	350 (370)	355 (282.5)	182, V=16938, P= 0.64
Age: Less than 1 year	420 (345)	480 (280)	27, V=393, P= 0.63	350 (315)	345 (247.5)	26, V=325, P=1.0
1-5 years	420 (400)	420 (330)	269, V=35540, P=0.72	315 (360)	360 (270)	276, V=37405, P=0.99
6-10 years	420 (330)	420 (322.5)	189, V=16941, P=0.43	360 (370)	360 (280)	184, V=16319, P=0.55
11+ years	400 (220)	420 (250)	43, V=842, P=0.60	300 (225)	280 (220)	43, V=969, P= 0.70
Dogs energy levels: High	540 (490)	420 (350)	198, V=17897, P=0.16	360 (390)	402.5 (225)	199, V=19107, P=0.55
Medium	420 (320)	420 (320)	294, V=42559, P=0.80	340 (345)	360 (270)	288, V=41173, P=0.88
Low	300 (292.5)	337.5 (232.5)	37, V=562, P=0.34	225 (260)	205 (195)	38, V=648, P=0.56
Relationship: Weak	420 (210)	420 (200)	14, V=109, P=0.63	250 (65)	300 (107.5)	13, V=51, P=0.16

Medium	420 (350)	420 (305)	263, V=33559, P=0.61	300 (285))	310 (210)	262, V=34740, P=0.75
Strong	420 (400)	420 (330)	253, V=31046, P=0.56	420 (420)	420 (290)	250, V=32886, P=0.27

Table S3: Multivariable logistic regression model results for dog's reduction of total weekly dog walking duration (compared to stayed the same or increased) during the first UK COVID-19 lockdown (March / April 2020) before the backward elimination was used to identify significant variables. The model was run on 486 observations, X^2 = 18.14, P=0.32, Pseudo R^2 (Cragg-Uhler) =0.053, AIC=588.23

**Variables (n)**	**Categories**	**Odds Ratio (95% Confidence Interval)**	**Z value**	***P***	**Reduced walking % (n)**	**Did not reduce/ increased walking % (n)**
Number of dogs (530)	1	1	-	-	16.8 (89)	39.8 (211)
	2	1.20 (0.75-1.93)	0.76	0.44	10.2 (54)	19.4 (103)
	3 or more	1.62 (0.89-2.97)	1.81	0.12	5.3 (28)	8.5 (45)
Dog size (526)	Small or toy	1	-	-	6.3 (33)	16.2 (85)
	Medium	1.13 (0.67-1.93)	0.35	0.81	14.6 (77)	27.2 (143)
	Large or giant	0.97 (0.56-1.70)	-0.4	0.88	11.4 (60)	24.3 (128)
Dog's age (530)	Under 1 years old	1	-		1.5 (8)	3.8 (20)
	1-5 years old	0.89 (0.34-2.31)	-0.49	0.81	17.2 (91)	33.8 (179)
	6-10 years old	0.93 (0.34-2.52)	-0.48	0.88	11.7 (62)	24.0 (127)
	10 +	0.48 (0.13-1.70)	-1.45	0.25	1.9 (10)	6.2 (33)
Dog's sex (530)	Male	1	-	-	17.7 (94)	35.6 (189)
	Female	0.97 (0.64-1.46)	-0.68	0.87	14.5 (77)	32.1 (170)
Dog energy levels (530)	Low	1	-	-	2.3 (12)	4.7 (25)

	Medium	0.75 (0.33-1.70)	-0.62	0.49	16.8 (89)	38.7 (205)
	High	0.73 (0.31-1.75)	-0.94	0.48	13.2 (70)	24.3 (129)
Relationship with dog (530)	Weak	1	-	-	0.8 (5)	1.9 (10)
	Medium	0.75 (0.33-1.70)	0.18	0.72	16.4 (87)	33.2 (176)
	Strong	1.46 (0.35-6.05)	0.38	0.6	15.1 (80)	32.6 (173)
Owner’s living arrangements (522)	Owner does not live alone	1	-	-	32.8 (171)	58.0 (303)
	Owner lives alone	1.71 (1.0-3.0)	1.78	0.05	6.5 (34)	9.6 (50)
Owner's age (528)	18-30	1	-	-	6.8 (36)	12.5 (66)
	30-50	1.29 (0.75-2.22)	0.9	0.36	15.5 (82)	27.7 (146)
	50+	0.68 (0.37-1.23)	-1.28	0.20	10.0 (53)	27.3 (144)
Owner's gender (527)	Man	1	-	-	2.8 (15)	7.8 (41)
	Woman	1.31 (0.63-2.73)	0.73	0.46	29.4 (155)	59.8 (315)
Owner’s education (527)	University degree	1	-	-	19.4 (102)	36.2 (191)
	Professional diploma	1.23 (0.72-2.13)	0.76	0.44	5.9 (31)	12.7 (67)
	A-level or equivalent	0.79 (0.40-1.54)	-0.69	0.49	3.2 (17)	8.9 (47)
	Below A-level or equivalent	1.20 (0.61-2.34)	0.53	0.59	4.0 (21)	9.7 (51)
Does the owner have a care plan (529)	No	1	-	-	19.1 (101)	43.5 (230)
	Yes	1.29 (0.84-1.99)	1.18	0.23	13.2 (70)	24.3 (128)
Symptoms of COVID-19 (529)	Owner or household members did not experience	1	-	-	28.2 (149)	60.1 (318)

	COVID-19 symptoms					
	Owner or household members did experience COVID-19 symptoms	1.26 (0.67-2.38)	0.84	0.40	4.2 (22)	7.6 (40)
Owner/household vulnerability to COVID-19 (529)	Owner or household members not described as vulnerable	1	-	-	22.9 (121)	53.3 (282)
	Owner or household member considered vulnerable	1.78 (1.11-2.86)	1.62	0.10	9.5 (50)	14.4 (76)

Table S4: Multivariable regression analysis of variables associated with duration of person's dog walking before the first UK COVID-19 lockdown (March / April 2020). The model is based on 511 observations, F(22) = 7.63, P = 0.00, Cragg-Uhler Pseudo-R^2 = 0.21, AIC = 396.60

	**Comparison category**	**Estimate**	**Standard Error**	**t value**	**p-value**
(Intercept)	2.22	0.16	14.22	< 2e-16	
Number of dogs: 2	Number of dogs: 1	0.02	0.04	0.42	0.67
Number of dogs: 3	Number of dogs:1	0.11	0.05	2.18	0.03
Size: medium	Size: small/toy	0.07	0.04	1.61	0.11
Size:large/ giant	Size: small/toy	0.06	0.04	1.52	0.13
Age: 1-5	Age: Under 1	-0.06	0.07	-0.78	0.43
Age: 6-10	Age: Under 2	-0.03	0.08	-0.38	0.71
Age: 10+	Age: Under 3	-0.05	0.09	-0.53	0.60
Dog's sex: male	Sex:male	-0.03	0.03	-0.98	0.33
Dog's energy: medium	Energy:low	0.12	0.07	1.78	0.08
Dog's energy: high	Energy:low	0.16	0.07	2.34	0.02
Relationship: medium	Relationship: weak	0.02	0.11	0.14	0.89
Relationship: strong	Relationship: weak	0.06	0.12	0.56	0.58
Owner lives alone	Owner does not live alone	0.12	0.04	2.81	0.01
Owner's age: 30-50	Owner's age: 18-30	0.10	0.04	2.31	0.02

Owner's age: 50+	Owner's age: 18-30	0.09	0.05	1.87	0.06
Owner's gender: female	Owner's gender: male	0.04	0.05	0.70	0.48
Owner's education: Professional	Owner's education: University Degree	-0.01	0.04	-0.14	0.89
Owner's education: A-levels or equivalent	Owner's education: University Degree	-0.18	0.05	-3.58	0.00
Owner's education: below A-level or equivalent	Owner's education: University Degree	0.01	0.05	0.29	0.77

Table S5. Multivariable logistic regression model results for person's reduction of total weekly dog walking duration (compared to stayed the same or increased) during the first UK COVID-19 lockdown (March / April 2020) before the backward elimination was used to identify significant variables. The model was run on 491 observations, X2 = 25.92, P=0.13, Pseudo R2 (Cragg-Uhler) =0.074, AIC=601.06.

**Variables (n)**	**Categories**	**Odds Ratio (95% Confidence Interval)**	**Z value**	**P**	**Reduced walking % (n)**	**Did not reduce/ increased walking % (n)**
Number of dogs (523)	1.00	1.00	-	-	13.4 (7)	40.0 (209)
	2.00	1.20 (0.75-1.93)	0.48	0.45	7.7 (40)	20.5 (107)
	3 or more	1.73 (0.96- 3.15)	2.08	0.07	5.0 (26)	8.2 (43)
Dog size (519)	Small or toy	1.00	-	-	5.8 (30)	15.8 (82)
	Medium	1.1 (0.65- 1.86)	0.56	0.73	11.6 (60)	28.3 (147)
	Large or giant	0.89 (0.51- 1.56)	-0.01	0.69	8.9 (46)	24.3 (126)
Dog's age (523)	Under 1 years old	1.00	-	-	1.3 (7)	3.4 (18)
	1-5 years old	0.79 (0.31- 2.03)	-0.05	0.62	13.4 (70)	35.2 (184)
	6-10 years old	0.79 (0.29- 2.10)	0.16	0.63	9.9 (52)	23.7 (124)

	10 +	0.39 (0.11- 1.39)	-0.99	0.15	1.3 (7)	6.3 (33)
Dog’s sex (523)	Male	1.00	-	-	14.3 (75)	36.1 (189)
	Female	0.87 (0.57- 1.31)	-0.50	0.50	11.7 (61)	32.5 (170)
Dog energy levels (523)	Low	1.00	-	-	2.1 (11)	4.8 (25)
	Medium	0.77 (0.34-1.75)	-0.35	0.54	14.7 (77)	38.2 (200)
	High	0.66 (0.28 -1.57)	-0.52	0.35	9.2 (48)	25.6 (134)
Relationship with dog (523)	Weak	1.00	-	-	0.6 (3)	1.9 (10)
	Medium	1.13 (0.28- 4.5)	0.14	0.86	11.7 (61)	35.4 (185)
	Strong	1.31 (0.33- 5.23)	0.57	0.71	13.8 (72)	31.4 (164)
Owner’s living arrangements (515)	Owner does not live alone	1.00	-	-	20.4 (105)	59.4 (306)
	Owner lives alone	1.63 (0.95-2.80)	2.10	0.08	5.6 (29)	9.5 (49)
Owner's age (521)	18-30	1.00	-	-	5.0 (26)	13.8 (72)
	30-50	1.26 (0.72- 2.22)	1.12	0.84	13.4 (70)	27.4 (143)
	50+	0.70 (0.37- 1.31)	-0.88	0.24	7.7 (40)	27.4 (143)
Owner's gender (521)	Man	1.00	-	-	1.7 (9)	8.1 (42)
	Woman	1.49 (0.68- 3.26)	1.65	0.32	24.4 (127)	60.6 (315)_
Owner’s education (522)	University degree	1.00	-	-	15 (78)	38.3 (199)
	Professional diploma	1.13 (0.65- 1.98)	0.60	0.66	5.4 (28)	11.7 (61)
	A-level or equivalent	0.69 (034- 1.40)	-1.15	0.31	2.5 (13)	9.4 (49)
	Below A-level or equivalent	1.0 (0.52- 1.95)	-0.39	0.99	3.3 (17)	9.4 (49)

Does the owner have a care plan (521)	No	1.00	-	-	14.4 (75)	44.44 (232)
	Yes	1.33 (0.86-2.04)	1.93	0.20	11.7 (61)	24.3 (127)
Symptoms of COVID-19 (523)	Owner or household members did not experience COVID-19 symptoms	1.00	-		22.6 (118)	320 (61.2)
	Owner or household members did experience COVID-19 symptoms	1.31 (0.70-2.46)	0.74	0.40	3.4 (18)	39 (7.5)
Owner/household vulnerability to COVID-19 (522)	Owner or household members not described as vulnerable	1.00	-	-	18.8 (98)	54.0 (282)
	Owner or household member considered vulnerable	1.50 (0.92-2.41)	1.52	0.10	7.3 (38)	14.8 (77)
